# Exploring intraspecific variation in salinity tolerance at germination and seedling development stages in *Camelina sativa*

**DOI:** 10.3389/fpls.2025.1713651

**Published:** 2025-11-24

**Authors:** Rossella Mastroberardino, Federica Zanetti, Andrea Monti

**Affiliations:** Department of Agriculture and Food Sciences (DISTAL), Alma Mater Studiorum, Università di Bologna, Bologna, Italy

**Keywords:** NaCl, salt stress, time-to-event analysis, stress tolerance index, cluster analysis, shoot and root length

## Abstract

**Introduction:**

*Camelina sativa* is a promising oilseed crop for cultivation on saline marginal lands due to its abiotic stress tolerance and low input requirements. However, intraspecific variation in salinity tolerance remains poorly understood.

**Methods:**

This study, through three sequential experiments, applied a screening framework integrating time-to-event modeling, stress tolerance indices (STIs), and multivariate clustering to dissect variation in salinity tolerance across early developmental stages. In experiment 1, two commercial varieties were germinated under a gradient from 0 to 300 mM of NaCl. In experiment 2, 57 camelina accessions were evaluated at 0 and 200 mM of NaCl for six germination indices (total germination, germination index, mean germination time, velocity coefficient, synchronization index, and normality rate) expressed as STIs, to quantify relative performance under salinity. In experiment 3, 13 representative accessions were assessed for seedling STIs (shoot length, main root length, lateral root length) under 0 and 200 mM of NaCl.

**Results and discussion:**

Time-to-event analysis revealed significant varietal differences in germination dynamics, with 200 mM identified as the optimal threshold for discriminating genotypic responses without complete germination inhibition. Most accessions retained ≥90% total germination under salinity, yet principal component analysis and hierarchical k-means clustering classified them into three phenotypic groups with distinct germination strategies. Salinity strongly reduced lateral root length (−90%), main root length (−80%), and shoot length (−30%), indicating altered biomass allocation in response to salt stress. Integration of germination clusters with seedling responses revealed three adaptive strategies: 1) high but delayed germination accompanied by strong seedling vigor, 2) low germination with intermediate seedling tolerance, and 3) high and rapid germination accompanied by poor seedling growth.

**Conclusions:**

These findings highlight salinity tolerance as a stage-dependent trait, underscoring the need for multistage phenotyping to guide breeding of *C. sativa* for saline environments.

## 1 Introduction

Soil salinization poses a growing threat to global agriculture, currently affecting more than 800 million hectares of land and over 20% of irrigated areas worldwide and causing an estimated economic damage of US$30 billion per year (FAO, 2024, 2021). As salinity increases, crop productivity is compromised through a complex interplay of osmotic stress, ion toxicity [primarily from sodium (Na^+^) and chloride (Cl^-^)], and nutrient imbalances, all of which disrupt physiological homeostasis and constrain development across the plant life cycle (Munns and Tester, 2008; Roy et al., 2014). Successful crop establishment is a critical determinant of final yield, especially under saline conditions; hence, seed germination and early seedling growth are considered some of the most sensitive developmental stages to salinity stress (Ali et al., 2014; Negacz et al., 2022; Zorb et al., 2019). Salinity reduces the osmotic potential of the soil solution, limiting water uptake and delaying or even preventing germination, while ion accumulation can impair enzymatic activity and cellular metabolism, undermining seedling vigor and establishment (Arzani, 2008; Valenzuela et al., 2022). Tolerance during germination does not necessarily translate into successful early growth (Javed et al., 2022; Munns and Gilliham, 2015), as seedlings must not only emerge but also develop functional roots and shoots capable of maintaining growth under osmotic and ionic stress. Therefore, both germination and early growth phases are key targets for identifying salt-tolerant genotypes as genetic resources for breeding programs. Developing varieties capable of maintaining productivity on marginal or degraded lands affected by salinity is essential for ensuring food security and sustainable agriculture (FAO, 2024; Hopmans et al., 2021).

Vegetable oils represent one of the most important agricultural commodities worldwide, with increasing demand for both food and feed purposes and, to a growing extent, for industrial applications such as biofuels and biopolymers (Lu et al., 2011; OECD/FAO, 2024). However, major oilseed crops, including soybean, sunflower, and rapeseed, are highly to moderately sensitive to salinity, with yield inhibition reported at electrical conductivity of 4–6 dS m^-^¹ (Butcher et al., 2016; Ceccoli et al., 2022; Wang et al., 2025). This restricts their suitability to be grown on salt-affected soils and highlights the need to explore alternative oilseeds that combine resilience with economic value. Among potential candidates, camelina [*Camelina sativa* (L.) Crantz], a re-emerging oilseed crop of the Brassicaceae family, has shown favorable traits. For instance, camelina germination remained largely unaffected up to ~8 dS m^-^¹, with a 25% decline only beyond ~35 dS m^-^¹, indicating greater salinity tolerance than several other minor oilseed crops (Matthees et al., 2018). Moreover, camelina is reported to have notable drought tolerance (Campbell et al., 2013; Čanak et al., 2020), suggesting an inherent capacity to withstand osmotic stress in saline soils, along with different agronomic advantages: a short and flexible life cycle (ranging from 85 to 220 days), low fertilizer and water demands, and compatibility with diverse cropping systems (Berti et al., 2016; Zanetti et al., 2021). Camelina seeds are rich in oil (30%–40%) and high in polyunsaturated fatty acids, suitable for applications ranging from advanced biofuels to many biobased products. The residual seed meal (~30% protein) offers additional value for animal feed or biorefinery uses (Alberghini et al., 2022; Mondor and Hernandez-Alvarez, 2022; Neupane et al., 2022). Despite these favorable traits, little is known about camelina intraspecific variation under saline conditions. Evaluating camelina response, starting from critical stages like germination and early growth, is therefore necessary to assess its potential as an oilseed crop for cultivation on salt-affected soils.

Germination and early-stage assays are widely employed in abiotic stress screening programs due to their speed, reproducibility, and suitability for high-throughput phenotyping. However, reliance on final germination percentage alone is insufficient to capture the full complexity of plant responses to salinity, since stress influences both the timing and the quality of germination (Hay et al., 2014). As a result, several methodologies have been developed to enhance the resolution of early-stage stress evaluation (McNair et al., 2012; Scott et al., 1984). To better characterize germination dynamics under stress conditions, time-to-event modeling approaches are increasingly used (Onofri et al., 2022; Romano and Stevanato, 2020). Among these, non-parametric models provide a flexible and robust framework for describing the cumulative germination process over time, without assuming predefined distributional forms (Onofri et al., 2010). These models allow estimating germination timing and probability parameters and appropriately handle censored data (seeds that fail to germinate within the observation period) (Ritz et al., 2013). This feature is particularly important under stress conditions, where delayed or inhibited germination is common and can otherwise bias results in classical analyses (Onofri et al., 2022). Although time-to-event approaches have been applied in germination studies of several species (Cristaudo et al., 2019; Effah and Clavijo McCormick, 2024; Puglisi et al., 2020), including camelina through halotime and halothermal models (Sanehkoori et al., 2023), their use remains limited compared with traditional static analyses.

In addition to the modeling approach, stress tolerance indices (STIs) remain a widely used conventional method for evaluating genotypic performance across both optimal (control) and stress conditions, particularly in large-scale screenings, enabling more reliable genotype ranking and minimizing the confounding effects of control condition variability (Aggarwal et al., 2024; Zhang et al., 2021). In the context of germination and seedling early development, STIs can be calculated for traits such as germination rate, mean germination time, shoot and root length, shoot and root weight, and vigor indices, offering an integrated view of physiological performance under salinity (Anwar et al., 2025; Li et al., 2020). When combined with multivariate statistical tools such as clustering or principal component analysis (PCA), these trait-based indices can reveal patterns of intraspecific variation and group accessions based on composite stress response profiles (Watson-Guido et al., 2025; Oyiga et al., 2016; Sivakumar et al., 2020; Zeng et al., 2002). This approach supports the identification of tolerant genotypes and facilitates their selection for breeding or further physiological investigation. Although camelina is frequently described as moderately salt-tolerant based on germination traits, most studies to date have focused on a limited number of commercial cultivars (Jovičić et al., 2025; Kukrić et al., 2023; Sanehkoori and Bakhshandeh, 2023; Sanehkoori et al., 2023).

To address these gaps, we integrated time-to-event germination modeling, stress tolerance indices, and multivariate analysis to characterize the intraspecific diversity of camelina under salinity stress during germination and seedling development and to assess whether germination traits can reliably predict subsequent performance.

## 2 Material and methods

To investigate camelina response to salinity at the early stage, three distinct experiments were conducted under controlled environment conditions at the Agricultural and Food Science Department of the University of Bologna, Italy (44.51°N, 11.41°E). In experiment 1, camelina germination response at increasing salinity levels was evaluated; in experiment 2, NaCl concentration identified as the most suitable for screening tolerance was used to explore camelina genotypic diversity; and finally, in experiment 3, seedling early development in response to salinity was surveyed.

### 2.1 Experiment 1: identification of the most suitable salinity level to discriminate response differences in camelina germination

#### 2.1.1 Trial setup and conduction

To define the most suitable salinity level for screening camelina accession tolerance at germination, the response of two commercial spring varieties (CCE 117, supplied by Camelina Company Espana, and Sonny, supplied by KWS Germany) to increasing concentrations of NaCl was tested. Seeds were incubated in petri dishes (120 mm diameter) containing a filter paper saturated with the assigned treatment solution. Seven solutions at increasing NaCl concentration were used as treatments: 0, 50, 100, 150, 200, 250, and 300 mM of NaCl, corresponding respectively to electrical conductivities (ECs) of 1.03, 4.12, 8.99, 13.98, 18.23, 24.34, and 26.83 dS m^−1^, measured with a portable conductometer (Hanna Instruments 98130). For each variety, three replicates of 50 seeds were compared for each treatment. Petri dishes were completely randomized in an incubator, set at 22°C with 12 h of light. Germinated seeds were recorded 1, 2, 3, and 6 days after sowing (DAS), counting seedlings with reported radicles at least 2 mm long, according to International Seed Testing Association (ISTA) (2009). Additionally, at the final count, 6 DAS, abnormal seedlings were distinguished among germinated ones, counting the seedlings with underdeveloped cotyledons and/or radicles.

#### 2.1.2 Statistical analysis of data

The germination rates of the two varieties in the tested treatments were fitted to a non-parametric time-to-event model (non-parametric maximum likelihood estimator, NPMLE) using RStudio (2023.03.0 + 386 “Cherry Blossom” Release for Windows), “drcte” package (version 1.0.30, Onofri, 2023). In order to calculate a *P*-value for the null hypothesis that the three curves are not significantly different, a permutation test was performed (*P* ≤ 0.05). From the obtained models, Wilcoxon scores, time to reach 50% of germinated seeds (*T*_50_), and standard errors of *T*_50_ were extracted for each variety in each treatment, as reported by Onofri et al. (2022). Finally, total germination and abnormality percentages were calculated as the ratio of germinated seeds at 6 DAS to the total sown seeds and of abnormal seedlings at 6 DAS to the total germinated seeds. Total germination and abnormality percentages were compared among the different NaCl concentrations in each variety using RStudio. After verification of the assumption of normality and homoscedasticity, ANOVA was performed (*P* ≤ 0.05). Tukey’s test was used to separate means, using the Sidak method to adjust the *P*-value for multiple comparisons using the “emmeans” package (version 1.10.0; Lenth, 2020) and “multcomp” package (version 0.1-10; Hothorn et al., 2008).

### 2.2 Experiment 2: camelina genotypic diversity in response to salinity at germination

#### 2.2.1 Trial setup and conduction

Seeds from 57 camelina accessions from different sources (public accessions, commercial materials, experimental lines) and geographical origins (Europe, Asia) were compared, as shown in **Supplementary Table S1** of the **Supplementary Materials**. The investigated lines were all spring type, except for line 56, a winter camelina variety from Poland (**Supplementary Materials, Table S1**). Seeds were incubated in petri dishes (120 mm diameter) containing a filter paper saturated with 200 mM of NaCl solution or pure water as a control, since this salinity level was selected as the most suitable concentration to discriminate salinity response in camelina at the germination stage, as a result of experiment 1. The average EC of saline and control solution was 18.74 and 1.03 dS m^−1^, respectively, measured with a portable conductometer (Hanna Instruments, US, mod. 98130). For each camelina accession, three replicates of 50 seeds were performed for each treatment. Petri dishes were completely randomized in the incubator, set at 22°C with 12 h of light. Germinated seeds were recorded 1, 2, 3, and 6 DAS, counting seedlings with reported radicles at least 2 mm long, according to International Seed Testing Association (ISTA) (2009). Additionally, at the final count 6 DAS, abnormal seedlings were distinguished among germinated seeds, counting the seedlings with underdeveloped cotyledons and radicles.

#### 2.2.2 Calculation of germination indices

To evaluate different aspects of germination dynamics in the camelina accessions, the following germination indices were calculated: total germination (TG, final proportion of germinated seeds), velocity coefficient (VC, overall germination speed), germination index (GI, weighted measure of early germination), mean germination time (MGT, average time to germination), germination synchrony index (GSI, temporal uniformity of germination), and normality rate (NR, proportion of normal seedlings among germinated seeds). Indices were calculated according to the following formulas:


$$TG\;\left(\%\right)=\frac{G6}{Ntot}\times 100$$

$$VC\;\left(DAS^{-1}\right)=\frac{G6}{\sum \left(Nt\times Dt\right)}$$

$$GI\;\left(Seed\;DAS^{-1}\right)=\;\sum \frac{Nt}{Dt}\;$$

$$MGT\;\left(DAS\right)=\;\frac{\;\sum \left(Nt\times Dt\right)}{Ntot}$$

$$GSI=\;\;\frac{\sum \left(Nt\times \left(Nt-1\right)\right)}{G6\times \left(G6-1\right)}$$

$$NR\;\left(\%\right)=\;\frac{Nnorm}{G6}\times 100$$

Where *G*6 is the total germinated seeds at the final count (6 DAS), *Ntot* is the number of sown seeds (50), *Nt* is the number of germinated seeds between the time count *t* − 1 and time count *t*, *Dt* is the number of DAS at which time count *t* was conducted, and *Nnorm* is the number of normal seedlings recorded at the final count (6 DAS). To better evaluate the response to salinity of each camelina accession, STIs were calculated as follows:


$$STI_{x}=\;\frac{X_{200}}{X_{0}}$$

Where STI*_x_* is the STI of the germination index *x*, *X*_0_ is the value of the germination index *x* under control conditions (0 mM of NaCl), and *X*_200_ is the value of the germination index *x* under saline conditions (200 mM of NaCl).

#### 2.2.3 Statistical analysis of data

Data were further analyzed using RStudio. Based on the STIs of the calculated germination indices, the tested camelina accessions were divided into three different clusters through hierarchical *k*-means clustering analysis, using the “factoextra” package (version 1.0.7; Kassambara and Mundt, 2020). To explore the coordination of germination indices, principal components analyses (PCAs) of STIs were conducted on all the camelina accessions and for each cluster separately, using the “factoextra” package (version 1.0.7; Kassambara and Mundt, 2020). STIs among clusters were compared using the FSA package (Ogle et al., 2020). Since normality and homoscedasticity assumptions were violated, the Kruskal–Wallis test followed by Dunn’s test with a Bonferroni correction at *P* ≤0.05 was applied.

### 2.3 Experiment 3: camelina genotypic diversity in response to salinity during seedling development

#### 2.3.1 Trial setup and conduction

Thirteen camelina genotypes, representative of the three clusters (7 for cluster 1, 3 for cluster 2, and 4 for cluster 3), as identified in experiment 2 described in Section 2.2, were selected to survey camelina response to salinity at seedling development. Seeds were incubated in transparent germination boxes (120 mm × 180 mm ×40 mm) containing a blue blotter germination paper substrate saturated with 200 mM of NaCl solution or pure water as a control. The average EC of the saline and control solution was 18.74 and 1.03 dS m^−1^, respectively, measured with a portable conductometer (Hanna Instruments, US, mod. 98130). For each camelina genotype, five replicates of 11 seedlings were compared in each treatment, in order to ensure a distance of 15 mm between seeds along the germination substrate. Boxes were completely randomized in the incubator, set at 22°C with 12/12 h light/dark. At 8 DAS, individual photos of each box were taken and analyzed using ImageJ v1.54 (National Institutes of Health, Bethesda, MD, USA) to measure the main root length, the lateral root length, and the shoot length of the seedlings. To evaluate the global early-stage performance of genotypes under saline and control conditions, the seedling vigor index (SVI) was calculated for each box as follows:


SVI=Germinability*(Shoot lenght+Main root lenght+Lateral root lenght)

Where germinability (%) was the average germination rate reached by each thesis 6 DAS in experiment 2, shoot length (mm) was the average shoot length 8 DAS in each box, and main root length (mm) and lateral root length (mm) were the average main and lateral root length 8 DAS in each box.

To better explore the response to salinity in camelina genotypes, STIs were calculated for the average main root length, the average lateral root length, the average shoot length, and SVI of each box, as follows:


$$STI_{x}=\;\frac{X_{200}}{X_{0}}$$

Where STI*_x_* is the STI of the parameter *x*, *X*_0_ is the value of the parameter *x* under control condition (0 mM of NaCl), and *X*_200_ is the value of the parameter *x* under saline condition (200 mM of NaCl).

#### 2.3.2 Statistical analysis of data

To highlight differences in seedling salt response among the clusters identified at the germination stage, STIs among cluster 1, 2, and 3 accessions were compared using the FSA package (Ogle et al., 2020) of RStudio. Since normality and homoscedasticity assumptions were violated, the Kruskal–Wallis test followed by Dunn’s test with a Bonferroni correction at *P* ≤0.05 was applied.

## 3 Results

### 3.1 Experiment 1: identification of the most suitable salinity level to discriminate response differences in camelina germination

**Figure 1:**
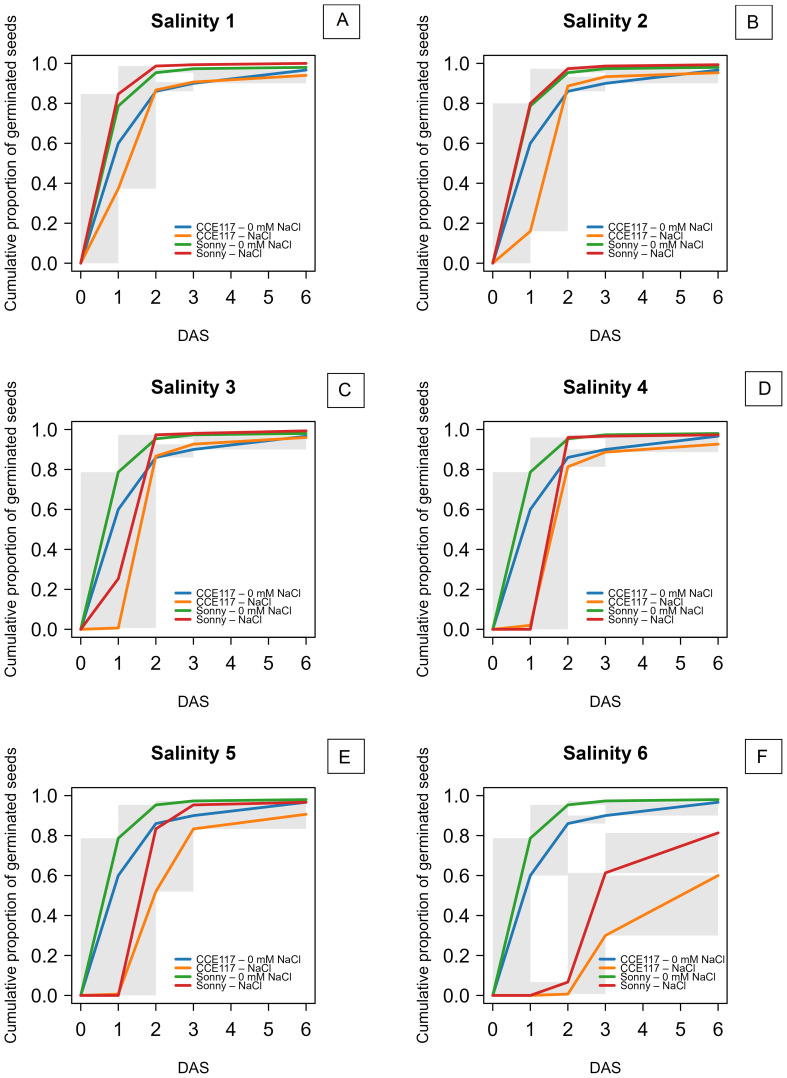
Non-parametric time-to-event curves for seed germination of CCE117 and Sonny at 0 mM of NaCl compared to 50 mM of NaCl **(A)**, 100 mM of NaCl **(B)**, 150 mM of NaCl **(C)**, 200 mM of NaCl **(D)**, 250 mM of NaCl **(E)**, 300 mM of NaCl **(F)**. The gray areas represent uncertainty due to censoring.

**Figure 2:**
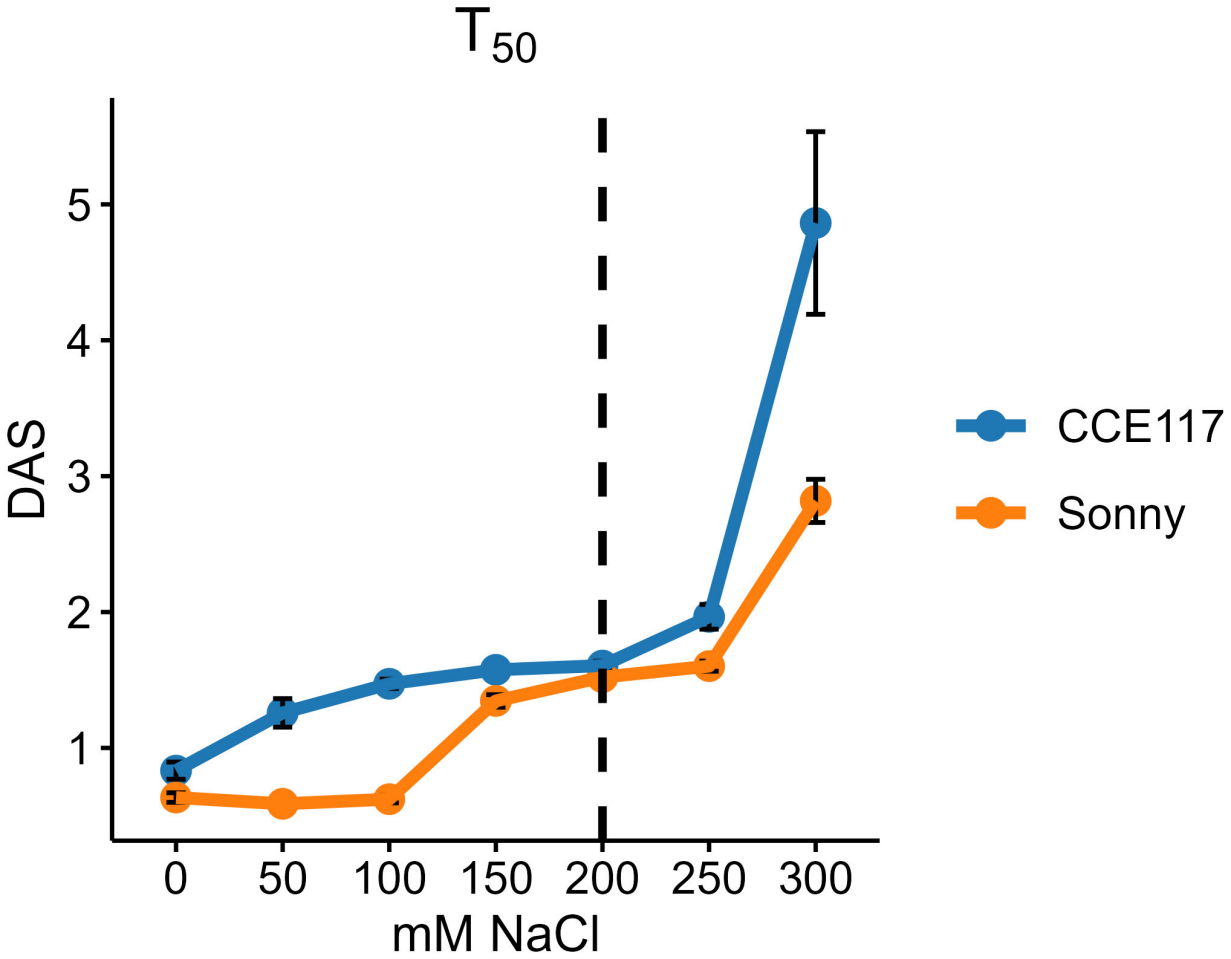
Time to reach 50% of total germinated seeds (*T*_50_) in CCE117 and Sonny at 0, 50, 100, 150, 200, 250, and 300 mM of NaCl. Error bars indicate standard errors. The black dotted line highlights the *T*_50_ under 200 mM of NaCl, where both varieties showed a plateau phase with similar values.

**Figure 3:**
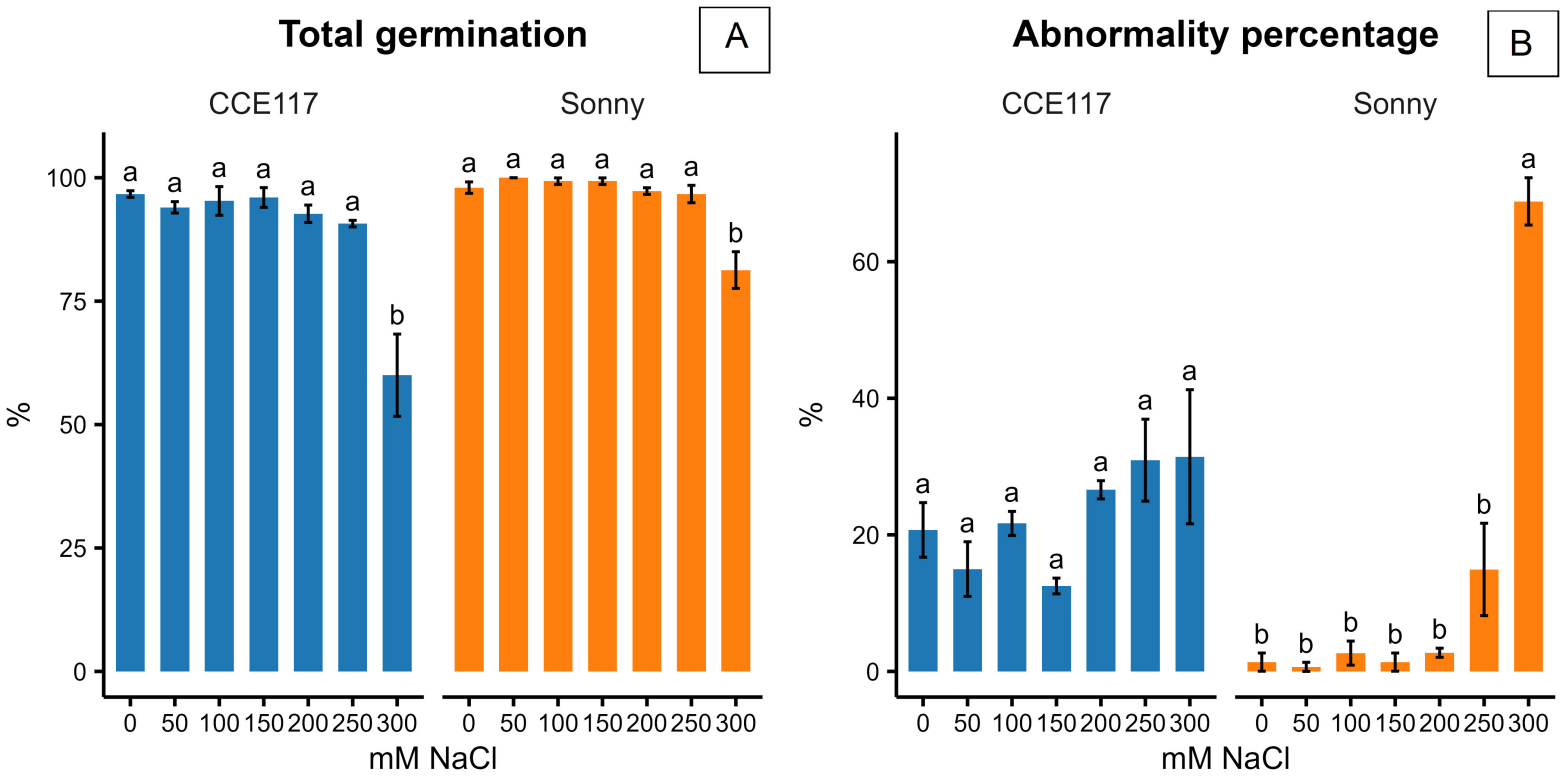
Total germination **(A)** and abnormality percentage on total germinated seeds **(B)** reported 6 DAS by the two tested camelina varieties at 0, 50, 100, 150, 200, 250, and 300 mM of NaCl. The error bars represent the standard errors. Separation of means was conducted separately for the two varieties. Different letters indicate significant means for *P* ≤0.05.

The germination of two commercial spring camelina varieties was evaluated at seven solutions with increasing NaCl concentrations, ranging from 0 to 300 mM. In **Figure 1**, the cumulative proportion of germinated seeds for both varieties at each salinity level, compared to 0 mM of NaCl, is presented over time, following the fitting of a non-parametric time-to-event model (**Table 1**). CCE117 exhibited overall lower germination than Sonny, both under control and saline conditions, as confirmed by the lower Wilcoxon scores for CCE117 (**Table 1**). At the first DAS, germination of CCE117 decreased in all the tested salinity levels compared to 0 mM of NaCl (**Figure 1A**), while Sonny began to show a noticeable decrease only above 150 mM of NaCl (salinity level 3, **Figure 1C**). However, at 2 and 3 DAS, the germination resulted in being slightly higher under saline conditions compared with the control, up to 150 mM of NaCl in CCE117 (**Figures 1A–C**) and 200 mM of NaCl in Sonny (**Figures 1A–D**). The probability of seed germination over the total time analyzed was compared among treatments using Wilcoxon scores (**Table 1**). Consistent with the germination observed at 1 DAS, a reduced Wilcoxon score compared to the control was detected from 50 mM of NaCl in CCE117 and from 150 mM of NaCl in Sonny (**Table 1**). At 50 and 100 mM of NaCl, Sonny germination was generally more probable than at 0 mM of NaCl (**Table 1**). Germination probability dropped significantly in both varieties at 200 mM of NaCl, the first salinity level where negative Wilcoxon scores were observed (**Table 1**). The time to reach 50% of total germination (*T*_50_) was extracted from the model for each treatment (**Table 1**, **Figure 2**). In CCE117, *T*_50_ gradually increased from 0 mM of NaCl, while in Sonny it remained stable at 0.6 DAS up to 100 mM of NaCl (**Figure 2**). At ~200 mM of NaCl, both varieties reached a plateau with similar *T*_50_ values (1.5–1.6 DAS), followed by a sharp increase at 300 mM of NaCl, more pronounced in CCE117 (4.8 DAS) than in Sonny (2.8 DAS) (**Figure 2**). Focusing on the final total germination, both varieties showed significantly reduced germination compared to the control, only at the highest salinity level of 300 mM of NaCl (**Figure 3A**). In CCE117, germination dropped from 97% to 60% from 0 to 300 mM, while in Sonny, it decreased from 98% to 81% (**Figure 3A**). Notably, CCE117 maintained a constant percentage of abnormal seedlings across treatments (approximately 20% of germinated seeds), whereas Sonny showed a sharp increase in abnormal seedlings at 300 mM of NaCl, reaching 56% of germinated seeds (**Figure 3B**).

**Table 1:** Wilcoxon score, time to reach 50% of the total germinated seeds (*T*_50_), and *T*_50_ standard error (SE_50_) extracted from non-parametric time-to-event curves of the two camelina varieties under increasing NaCl concentrations (0, 50, 100, 150, 200, 250, and 300 mM of NaCl).

Variety	Salinity (mM of NaCl)	Wilcoxon score	*T*_50_ (DAS)	SE_50_ (DAS)
CCE117	0	47.74	0.833	0.063
	50	22.32	1.258	0.105
	100	0.89	1.472	0.034
	150	−18.28	1.575	0.020
	200	−23.21	1.606	0.032
	250	−52.57	1.965	0.091
	300	−118.8	4.863	0.672
Sonny	0	79.33	0.635	0.035
	50	90	0.589	0.023
	100	83.22	0.623	0.027
	150	21.16	1.346	0.046
	200	−9.44	1.521	0.013
	250	−21.18	1.602	0.037
	300	−101.16	2.818	0.159
Permutation *P*-value	0.005			

In the last line, the permutation *P*-value of the non-parametric time-to-event model fitting is reported.

### 3.2 Experiment 2: camelina genotypic diversity in response to salinity at germination

**Figure 4:**
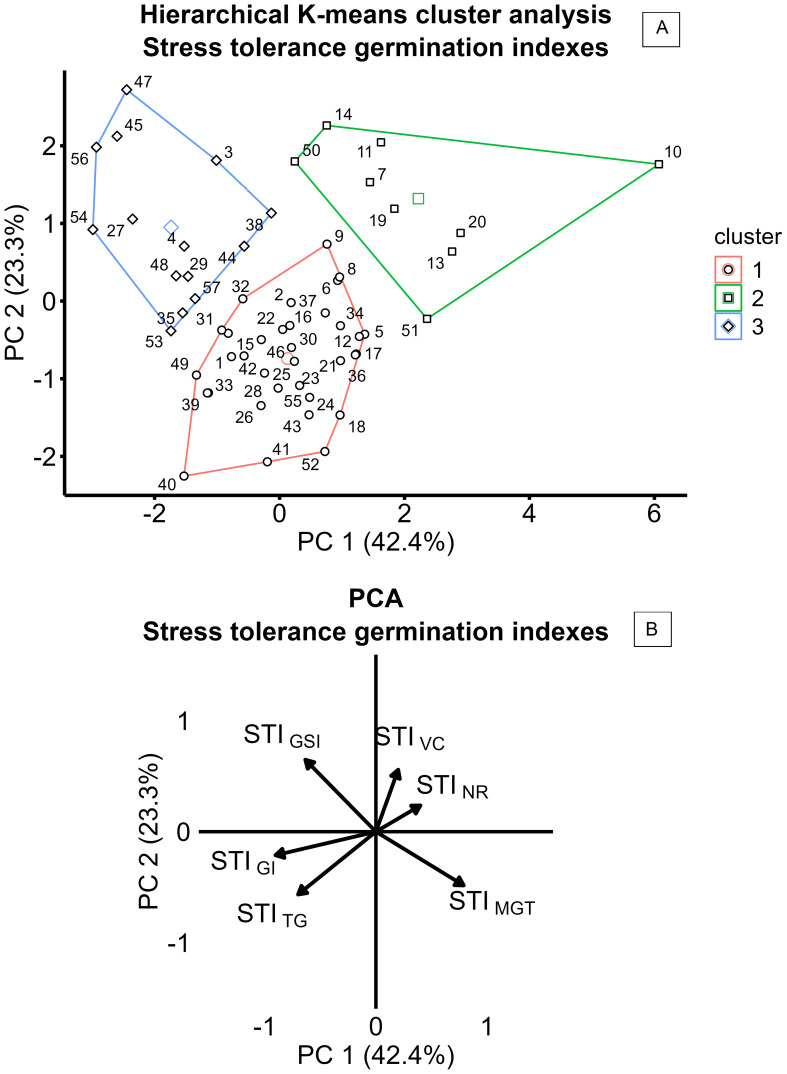
Hierarchical *k*-means cluster analysis of 57 camelina accessions **(A)**, based on principal component analysis (PCA) of stress tolerance indices (STIs) **(B)**, calculated as the ratio of the value reported at 200 mM of NaCl to the value reported at 0 mM of NaCl for TG (total germination), NR (normality rate), GI (germination index), VC (velocity coefficient), GSI (germination synchrony index), and MGT (mean germination time).

**Figure 5:**
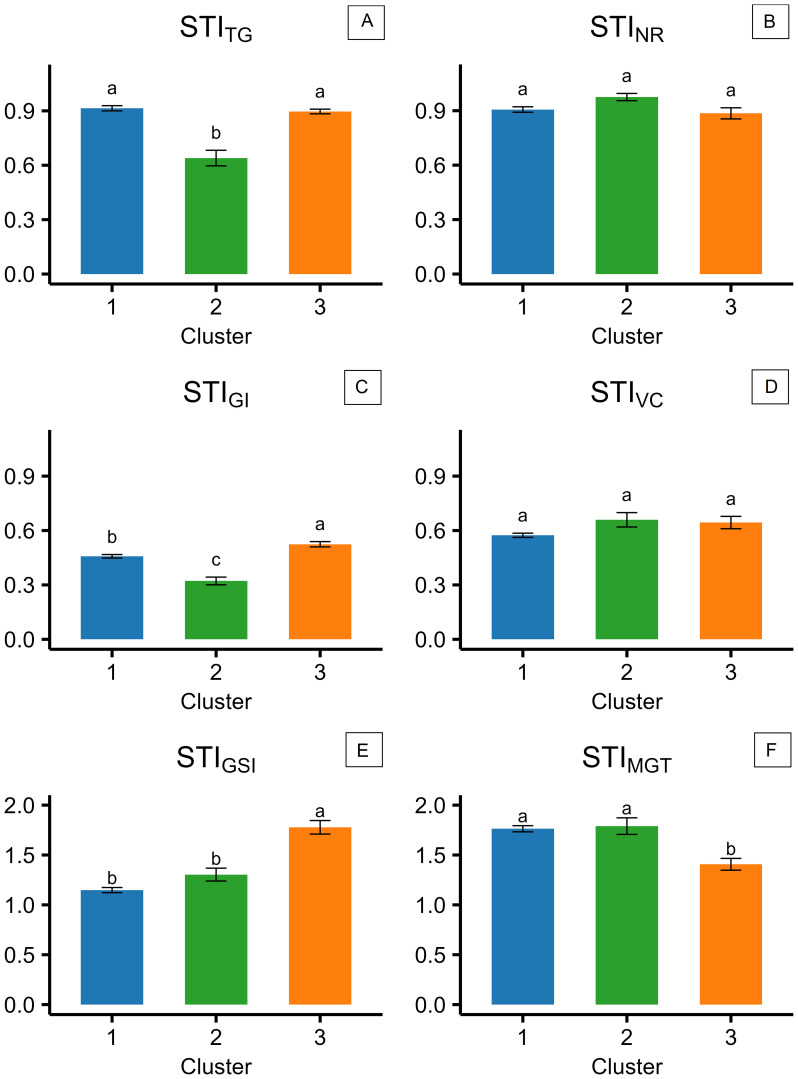
Stress tolerance indices (STIs), calculated for the three different clusters of camelina accessions as the ratio of the value reported at 200 mM of NaCl to the value reported at 0 mM of NaCl for TG (total germination) **(A)**, NR (normality rate) **(B)**, GI (germination index) **(C)**, VC (velocity coefficient) **(D)**, GSI (germination synchrony index) **(E)**, and MGT (mean germination time) **(F)**. The error bars represent the standard errors. Different letters indicate significant means for *P* ≤0.05.

**Figure 6:**
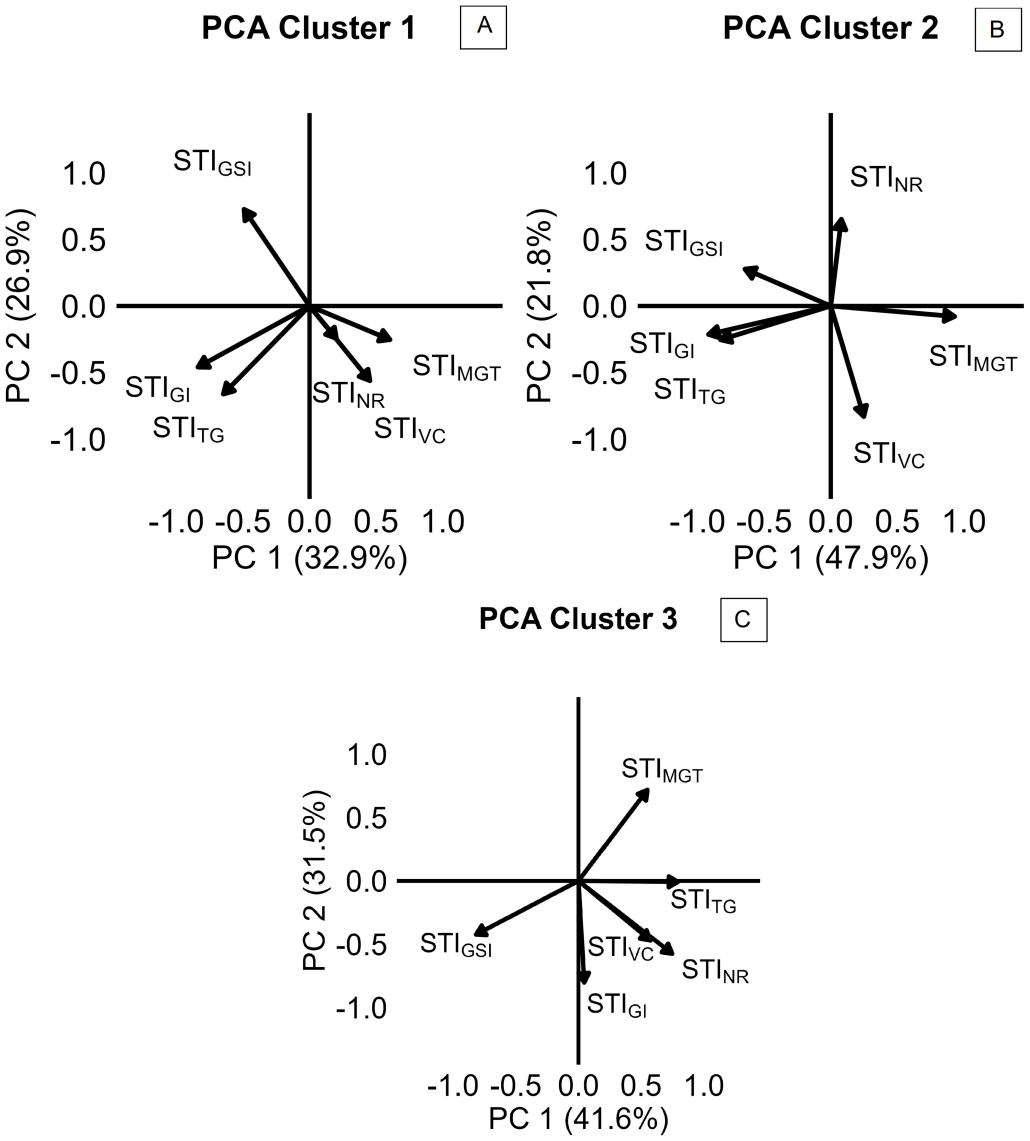
Principal component analysis (PCA) of stress tolerance indices (STIs), reported by cluster 1 **(A)**, cluster 2 **(B)**, and cluster 3 **(C)** of camelina accessions, calculated as the ratio of the value reported at 200 mM of NaCl to the value reported at 0 mM of NaCl for TG (total germination), NR (normality rate), GI (germination index), VC (velocity coefficient), GSI (germination synchrony index), and MGT (mean germination time).

Variation in salinity response among the 57 camelina genotypes was explored through stress tolerance indices of total germination, velocity coefficient, germination index, mean germination time, germination synchrony index, and normality rate (STI_TG_, STI_VC_, STI_GI_, STI_MGT_, STI_GSI_, and STI_NR_). PCA based on STIs is presented in **Figure 4B**. The first two principal components accounted for 65.7% of the total variance, with PC1 explaining 42.4% and PC2 explaining 23.3% (**Figure 4B**). PC1 was primarily associated with positive contributions from STI_MGT_ and negative contributions from STI_GI_ and STI_TG_, reflecting the contrast between germination timing traits, expected to increase under saline conditions, and germination rate and uniformity traits, expected to decrease under salinity (**Figure 4B**). PC2 was positively influenced by STI_GSI_ and STI_VC_ and negatively influenced by STI_TG_ and STI_MGT_, suggesting more complex dynamics in the salinity stress response (**Figure 4B**). Using hierarchical *k*-means cluster analysis, three distinct clusters of camelina accessions were identified based on PC1 and PC2 (**Figure 4A**). Clusters 1, 2, and 3 consisted of 32, 9, and 14 genotypes, respectively (**Figure 4A**). Cluster 1 exhibited lower PC2 scores, indicating a greater delay in germination under salinity expressed by higher STI_MGT_, while still maintaining a high STI_TG_ (**Figures 4A, B**). Cluster 2 showed higher PC1 values, representing accessions with lower stress tolerance across both germination timing and performance indices (**Figures 4A, B**). Cluster 3 had lower PC1 scores compared to cluster 2, suggesting better maintenance of STI_TG_ and STI_GI_ under stress (**Figures 4A, B**). These findings were further supported by the comparison of STIs across the three clusters, as shown in **Figure 5**. In all clusters, the analyzed STIs were below 1, except for STI_GSI_ and STI_MGT_ (**Figure 5**). This suggests increased synchronization of germination under saline conditions, indicated by STI_GSI_ >1, and an increase in germination time, STI_MGT_ >1 (**Figures 5E, F**). Thus, overall, a decline in both germination performance and speed was observed under the tested salt concentration compared to the control (**Figure 5**). STI_TG_ was higher in clusters 1 and 3, which were both able to maintain an STI_TG_ of 0.90, compared to cluster 2, where STI_TG_ was 0.62 (**Figure 5A**). Cluster 3 recorded the highest STI_GI_ (0.51), followed by cluster 1 (0.46), while cluster 2 had the lowest value (0.32) (**Figure 5C**). Moreover, cluster 3 reported the highest STI_GSI_ (1.76) and the lowest STI_MGT_ (1.38), while clusters 1 and 2 showed no significant differences in STI_MGT_, both averaging 1.75 (**Figures 5E, F**). No significant differences were observed across clusters for STI_VC_ and STI_NR_, which showed average values of 0.62 and 0.90, respectively (**Figures 5B, D**). Within-cluster PCA revealed distinct trait coordination profiles across the three groups (**Figure 6**). In cluster 1 (**Figure 6A**), STI_TG_, STI_GSI_, and STI_GI_ loaded negatively on PC1, while STI_MGT_ and STI_VC_ loaded positively. In cluster 3 (**Figure 6C**), PC1 was driven by positive loadings of STI_TG_, STI_NR_, STI_MGT_, and STI_VC_. Cluster 2 (**Figure 6B**) showed a unique loading pattern, where STI_NR_ loaded on PC2 independently from other STIs loading on PC1.

### 3.3 Experiment 3: camelina genotypic diversity in response to salinity during seedling development

**Figure 7:**
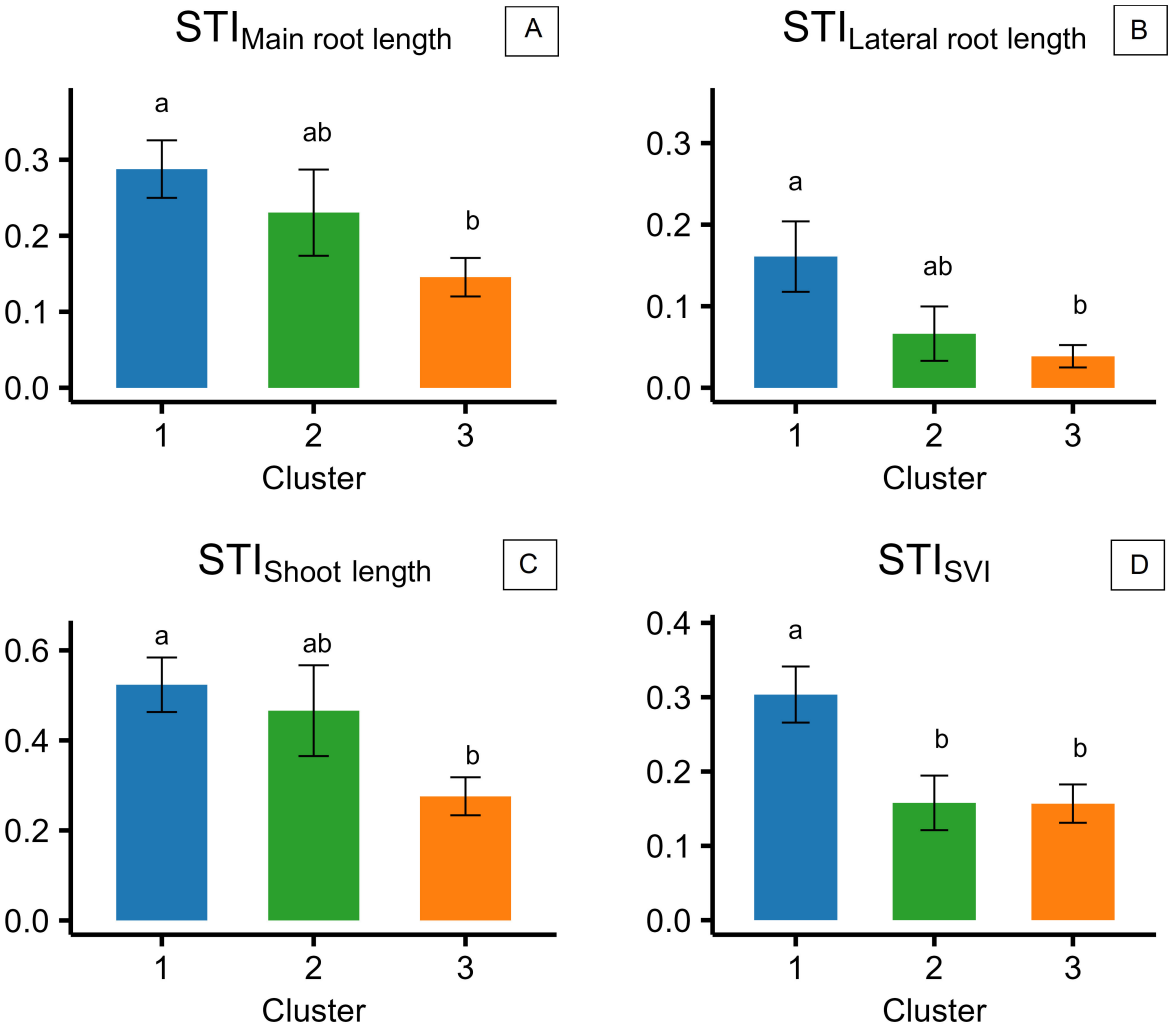
Stress tolerance indices (STIs), calculated for the three different clusters of camelina accessions as the ratio of the value reported at 200 mM of NaCl to the value reported at 0 mM of NaCl for main root length **(A)**, lateral root length **(B)**, shoot length **(C)**, and seedling vigor index (SVI) **(D)**. The error bars represent the standard errors. Different letters indicate significant means for *P* ≤0.05.

STI_Main root length_, STI_Lateral root length_, and STI_Shoot length_ showed a similar trend among the accessions of the three clusters highlighted in experiment 2, with accessions of cluster 1 showing higher STIs than those of cluster 3 (**Figures 7A–C**). Accessions of cluster 2 had intermediate values, which were not significantly different from either cluster 1 or 3 (**Figures 7A–C**). Generally, shoot length resulted to be the least impacted morphological trait by salinity, with STI ratios of 0.53, 0.47, and 0.24, respectively, in clusters 1, 2, and 3 (**Figure 7C**). Lateral root length was the most impacted by salinity, with STI ratios of 0.17, 0.07, and 0.04 in clusters 1, 2, and 3, respectively (**Figure 7B**), while main root length had STI ratios of 0.29, 0.23, and 0.15 in clusters 1, 2, and 3 (**Figure 7A**). STI_SVI_ was higher in cluster 1 (0.31) compared to clusters 2 and 3, with no significant differences and with an average value of 0.16 (**Figure 7D**).

## 4 Discussion

The present results provide new insights into camelina responses to salinity at germination and early seedling development stage, revealing both its overall resilience and substantial intraspecific diversity. In experiment 1, both tested commercial camelina varieties maintained high final germination rates (≥90%) under a broad range of salinities (0 to 250 mM of NaCl; **Figure 3A**), reflecting notable tolerance to salt stress in comparison to other glycophytic members of the Brassicaceae family. For example, germination in *Brassica napus* and *B. juncea* is commonly already halved at NaCl concentrations between 160 and 250 mM (Damalas and Koutroubas, 2025; Phour and Sindhu, 2020; Wang et al., 2022; Wu et al., 2019). However, previous studies on camelina reported contrasting results: some registered 50% germination reduction at 150–200 mM of NaCl (Jovičić et al., 2025; Kukrić et al., 2023; Sanehkoori and Bakhshandeh, 2023; Sanehkoori et al., 2023), while others observed responses more comparable to the present findings with germination remaining between 75% and 80% under 200–250 mM of NaCl (Bakhshandeh et al., 2025; Khalid et al., 2015). Since each of these previous investigations assessed only a limited number of lines, such discrepancies likely reflect substantial intraspecific variability. Reliance on a narrow genetic base may have underestimated camelina’s potential for salt tolerance, underscoring the need for broader phenotypic screening to identify genotypes better suited to saline environments. A more comprehensive screening approach, common to major crops such as *Hordeum vulgare*, *Sorghum bicolor*, *Helianthus annuus*, and *B. napus*, has proven effective in identifying tolerant accessions (Cope et al., 2022; Ding et al., 2018; Li et al., 2020; Wu et al., 2019). As supported by multiple germination parameters calculated in experiment 1, 200 mM of NaCl was selected as the most suitable concentration for screening salinity tolerance in camelina and then applied in the subsequent experiments. Wilcoxon scores for both camelina varieties became negative at 200 mM (**Table 1**), indicating that cumulative germination performance was measurably impacted at this concentration. *T*_50_ values had already begun to increase at lower concentrations, but 200 mM marked a plateau phase, a clear sign of sustained physiological response to salinity. At higher concentrations, both *T*_50_ and abnormality rates rose sharply (**Figure 2**). Thus, 200 mM represents a stressful, but non-lethal concentration, ideal for discriminating between tolerant and sensitive genotypes. A similar concentration was selected as optimal for screening accessions of *B. napus* by Wu et al. (2019) and of *B. juncea* by Aggarwal et al. (2024), supporting the relevance of this threshold across Brassicaceae. Consistently, previous time-to-event modeling of camelina germination under salinity stress (Sanehkoori et al., 2023) confirmed that germination is still maintained at 200 mM of NaCl at optimal temperatures, thereby validating its use as a stressful but non-lethal concentration for screening purposes. On this basis, experiment 2 screened 57 camelina accessions to evaluate germination responses under salinity. The results confirmed that camelina germination is relatively robust, with most accessions maintaining STI_TG_ approximately 0.9 at 200 mM of NaCl (**Figure 5A**), comparable to *H. annuus* (Ding et al., 2018), but superior to *B. napus*, which dropped to 0.5 under the same salt concentration (Wu et al., 2019). Camelina seeds tended to delay germination under stress, as indicated by higher STI_MGT_ (**Figure 5F**) than *Carthamus tinctorius* or *H. annuus* (Kaya et al., 2019), suggesting a protective adjustment mechanism. Crucially, this delay was not associated with elevated abnormality rates (**Figure 5B**), and synchronization even improved (STI_GSI_ > 1; **Figure 5E**), indicating that camelina maintained both viability and uniformity, valuable traits for consistent seedling establishment in saline soils. Experiment 3 evaluated whether germination patterns translate into later performance at the seedling stage. Camelina seedlings exhibited differential sensitivity to salinity across morphological traits, with shoot length being the least affected (mean STI = 0.41), followed by main root length (0.25), and lateral root length (0.09). This pattern suggests that, under salt stress, camelina prioritizes shoot elongation, supporting early photosynthesis and aboveground establishment even at the cost of reduced root expansion. Such a strategy may provide short-term benefits in mildly saline soils but could limit water and nutrient uptake under prolonged stress, as lateral roots, being more metabolically active and in direct contact with the saline solution, appear more susceptible to growth inhibition. However, studies conducted on A. thailliana reported a root growth stimulation under salinity (Ambastha et al., 2020; Arif et al., 2019). Contrasting results have been reported for camelina root length responses to ~200 mM of NaCl: Kukrić et al. (2023) observed stimulation in Serbian cultivars, whereas Berti et al. (2025) found inhibition in the spring line C046 and the winter line Joelle. Both studies applied comparable methodologies, confirming again a relevant genotypic effect in camelina response to salt stress. By integrating the cluster assignments obtained from multivariate analysis of germination traits in experiment 2 with the seedling responses measured in experiment 3, three distinct phenological profiles were delineated (**Figures 4**-**7**). Cluster 1 combined high total germination with slower germination processes, suggesting a conservative but effective strategy under salt stress. PCA analysis within this cluster (**Figure 6A**) revealed unusual coordination: STI_MGT_ and STI_VC_ loaded positively on PC1, an unexpected pattern since longer germination time typically associates with a lower velocity coefficient. This may reflect a two-phase germination dynamic: a delayed onset that allows seeds to adjust osmotically, followed by a rapid and synchronized completion. Such behavior could explain why these accessions, despite delayed emergence, ultimately achieved robust germination and high synchronization. Consistently, in experiment 3, cluster 1 accessions exhibited the highest STIs for main root length, lateral root length, shoot length, and SVI, confirming that germination delay did not compromise post-emergence development (**Figure 7**). Rather, the high seedling STIs observed in cluster 1 support the earlier findings: an initial germination delay may serve as an osmotic adjustment phase, followed by vigorous seedling growth. Notably, the commercial variety CCE117 (genotype 55; **Supplementary Materials, Table S1**) was assigned to cluster 1 (**Figure 4**), consistent with its behavior in experiment 1, where it showed increases in *T*_50_ even at low salinity levels (**Figure 2**) and maintained low abnormalities across all treatments (**Figure 3B**). This suggests that its stress-induced delay played as an osmotic adjustment, reducing ion toxicity and preserving seedling integrity, in line with reports that delayed germination in *B. napus* correlated with higher salt tolerance (Zhu et al., 2024). Cluster 2 showed generally poor germination performance and speed (**Figure 4**). PCA within this cluster revealed a separation of seedling abnormality (STI_NR_) from other germination traits, loading independently on PC2 (**Figure 6B**). This decoupling suggests that certain accessions may maintain post-emergence integrity despite failing to germinate efficiently, possibly due to resilience mechanisms activated later in development. Experiment 3 confirmed this interpretation, as these accessions, despite poor germination (**Figure 5**), were not the most affected by salinity at the seedling stage (**Figure 7**). This suggests that in these accessions, improved ion regulation, osmotic adjustment, or more efficient use of seed reserves may be expressed primarily during seedling growth, allowing tolerance to manifest after emergence. These findings highlight a key limitation in using germination as the only screening criterion for salt tolerance. Incorporating post-emergence traits into early-stage selection strategies is essential to avoid discarding genotypes with true adaptive potential. Cluster 3 genotypes adopted a different strategy: fast, uniform, and high final germination (**Figure 4**). PCA within this cluster showed strong positive loadings of STI_TG_, STI_GSI_, STI_VC_, and STI_NR_ on PC1, indicating a tight coordination between germination performance, speed, and seedling quality (**Figure 6C**). These accessions likely possess effective osmotic adjustment and membrane stability, enabling rapid water uptake. However, data from experiment 3 revealed that, despite rapid and uniform germination (**Figure 5**), cluster 3 accessions performed poorly at seedling development (**Figure 7**). Their early vigor under salinity was not sustained beyond germination, suggesting a trade-off between speed and resilience. Rapid mobilization of seed reserves may leave seedlings vulnerable to ionic and osmotic injury during elongation. The commercial variety Sonny (genotype 57; **Supplementary Materials, Table S1**) was assigned to this group (**Figure 4**), consistent with its behavior in experiment 1, where it showed mild germination stimulation at 50 mM, stable *T*_50_ up to 150 mM (**Table 1**), but a sharp increase in abnormalities at 300 mM (**Figure 3B**). This strategy resembles those observed in sensitive barley genotypes, where accelerated germination under salinity was linked to ion accumulation and seedling injury (Zhang et al., 2010). This strategy may be better suited to environments requiring quick stand establishment, such as short-season systems or transient salinity, but not for long-term survival under salt stress. Commonly, high germination under stress is considered a tolerance trait, while our results suggest that in camelina, this assumption does not always hold: accessions that germinated rapidly and uniformly (cluster 3, e.g., Sonny) failed to sustain vigor at the seedling stage, whereas those with slower but complete germination (cluster 1, e.g., CCE117) developed stronger seedlings. A comparable pattern was reported in El-Katony and El-Moghrbi (2025), where a salt-tolerant cultivar of *H. vulgare* exhibited slower germination than a sensitive one. These findings indicate that delayed germination may function as an adaptive strategy, allowing osmotic adjustment and reducing ion toxicity prior to emergence, and may therefore provide a more reliable predictor of salt tolerance than final germination alone. A recent large-scale study by Luo et al. (2020), focused on germination traits and seedling biomass production, evaluated 211 camelina accessions under 100 mM of NaCl integrating genome-wide association studies (GWAS). In Luo et al. (2020), genotypes Cs083, Cs092, Cs095, Cs101, Cs103, Cs151, Cs193, Cs205, and Cs233 correspond to genotypes from 45 to 53 in the present study (**Supplementary Materials, Table S1**), enabling a direct comparison under moderate (100 mM) and severe (200 mM) salinity. Luo et al. (2020) provided valuable genomic insights, and the present study may complement that work by characterizing germination dynamics under stronger salinity. Notably, the overlapping accessions showed different response patterns depending on stress intensity, which can be further understood through the cluster framework established here. For example, Cs151 and Cs193, assigned to cluster 2, maintained high germination and seedling weights at 100 mM but exhibited sharp reductions in germination at 200 mM, reflecting tolerance that becomes constrained from germination under stronger stress. Cs092, Cs103, and Cs205, assigned to cluster 1, showed stable germination across both studies: they had intermediate seedling weights at 100 mM, while at 200 mM, they expressed the strongest seedling elongation, consistent with their conservative but resilient strategy. In contrast, Cs083, Cs095, Cs101, and Cs233 were placed in cluster 3, yet their behavior was more variable. Cs083 and Cs095 germinated well at 100 mM but produced low seedling weights, resembling the weak vigor observed here under 200 mM, whereas Cs101 and Cs233 performed relatively well at 100 mM, suggesting that moderate stress may not be sufficient to expose their vulnerability. Together, these cross-study comparisons illustrate that tolerance classifications can shift with stress severity, reinforcing the need to evaluate both moderate and severe salinity levels when characterizing intraspecific diversity. Overall, the present study provided a more comprehensive framework for early-stage camelina selection under salinity by integrating germination dynamics with seedling vigor traits. Specifically, slower germination coupled with higher or intermediate seedling stress tolerance indices is suggested to be a more informative indicator of resilience than total emergence alone. To translate these insights into breeding gains, full-cycle, multi-environment validation is required: lines from contrasting clusters should be evaluated across the complete crop cycle to rigorously test the stability and predictive reliability of the cluster assignments. This will ensure that the proposed framework scales from controlled assays to field performance and can be deployed for parental selection and early-generation screening in saline environments.

## 5 Conclusions

The present study evaluated salinity tolerance in camelina during germination and seedling development, integrating time-to-event modeling, STIs, and clustering analysis. Two main conclusions emerged:

- Two hundred millimolars of NaCl is the most suitable concentration for germination and seedling development in camelina genotype screening programs. Based on germination timing (*T*_50_), Wilcoxon scores, and seedling abnormality rates, 200 mM NaCl was identified as a stress level that significantly impacted germination timing and performance without inducing lethal effects. This concentration successfully differentiated sensitive and tolerant genotypes and aligns with thresholds used in related Brassicaceae crops, supporting its use in high-throughput phenotyping protocols.
- Three distinct salinity response strategies were identified based on germination dynamics and seedling development: i) delayed germination and strong seedling vigor, suggesting a protective strategy suitable for prolonged saline exposure; ii) delayed and poor germination but intermediate seedling tolerance, suggesting latent post-emergence resilience; and iii) rapid and uniform germination but low seedling vigor, vulnerable to sustained stress.

These findings highlight that, in camelina, high final germination alone is not a reliable indicator of overall salt tolerance, whereas delayed but successful germination may provide a more robust indicator of adaptive potential. To validate the predictive value of these early-stage traits, future work should extend to later developmental phases and assess performance across the full growth cycle to evaluate the stability and predictive reliability of cluster assignments. Developing integrated methodologies that capture tolerance across multiple stages will enhance selection accuracy and reveal the physiological diversity needed to guide breeding for saline environments.

## Data Availability

The raw data supporting the conclusions of this article are available here: https://doi.org/10.5281/zenodo.17526004.
