# Supplementary material for: Exploring intraspecific variation in salinity tolerance at germination and seedling development stages in Camelina sativa
Source: Front Plant Sci. 2025 Nov 24;16:1713651. doi: 10.3389/fpls.2025.1713651 (PMC12683665; doi:10.3389/fpls.2025.1713651)
Supplement: Supplementary file 1 [file Table1.docx]

Supplementary Material

**Tab. 1** For each of the 57 camelina accessions, analyzed in the germination trial to explore diversity in response to salinity, are reported: the line number assigned for the present study, the UNT Code assigned within the HORIZON 2020 UNTWIST project, the geographical origin, the provider, the source and the ecotype. CCE Camelina Company Espana (ES), INRA Institut National de Recherche pour l'Agriculture, l'Alimentation et l'Environnement (FR), BOKU University of Natural Resources and Life Sciences Vienna (AU), UNIBO University of Bologna (IT), USDA United States Department of Agriculture (USA), UPP Poznań University of Life Sciences (PL), KWS KWS SAAT SE & Co. KGaA (DE), S Spring, W Winter, NA Not Avaliable.

| **Line** | UNT Code | **Geographical origin** | **Provider** | **Source** | **Ecotype** |
| --- | --- | --- | --- | --- | --- |
| 1 | UNT1 | Spain | CCE | CCE breeding program | S |
| 2 | UNT 2 | Spain | CCE | CCE breeding program | S |
| 3 | UNT 3 | Spain | CCE | CCE breeding program | S |
| 4 | UNT 4 | Spain | CCE | CCE breeding program | S |
| 5 | UNT 5 | Spain | CCE | CCE breeding program | S |
| 6 | UNT 6 | Spain | CCE | CCE breeding program | S |
| 7 | UNT 7 | Spain | CCE | CCE breeding program | S |
| 8 | UNT 8 | Spain | CCE | CCE breeding program | S |
| 9 | UNT 9 | Spain | CCE | CCE breeding program | S |
| 10 | UNT 10 | Spain | CCE | CCE breeding program | S |
| 11 | UNT 11 | Spain | CCE | CCE breeding program | S |
| 12 | UNT 12 | Spain | CCE | CCE breeding program | S |
| 13 | UNT 13 | Spain | CCE | CCE breeding program | S |
| 14 | UNT 14 | Spain | CCE | CCE breeding program | S |
| 15 | UNT 15 | Spain | CCE | CCE breeding program | S |
| 16 | UNT 16 | Spain | CCE | CCE breeding program | S |
| 17 | UNT 18 | France | INRAE | Public accession | S |
| 18 | UNT 19 | Poland | INRAE | Public accession | S |
| 19 | UNT 20 | Unknown | INRAE | Public accession | S |
| 20 | UNT 21 | Poland | INRAE | Public accession | S |
| 21 | UNT 22 | Former Yugoslavia | INRAE | Public accession | S |
| 22 | UNT 23 | Germany | INRAE | Public accession | S |
| 23 | UNT 24 | Russia | INRAE | Public accession | S |
| 24 | UNT 25 | Russia | INRAE | Public accession | S |
| 25 | UNT 26 | Russia | INRAE | Public accession | S |
| 26 | UNT 27 | Russia | INRAE | Public accession | S |
| 27 | UNT 28 | Russia | INRAE | Public accession | S |
| 28 | UNT 29 | Russia | INRAE | Public accession | S |
| 29 | UNT 30 | Russia | INRAE | Public accession | S |
| 30 | UNT 31 | Germany | INRAE | Public accession | S |
| 31 | UNT 32 | Denmark | INRAE | Public accession | S |
| 32 | UNT 33 | Unknown | INRAE | Public accession | S |
| 33 | UNT 34 | Austria | BOKU | Public accession | S |
| 34 | UNT 35 | Hungary | BOKU | Public accession | S |
| 35 | UNT 37 | China | BOKU | Public accession | S |
| 36 | UNT 38 | Kyrgyzstan | BOKU | Public accession | S |
| 37 | UNT 39 | Russia | BOKU | Public accession | S |
| 38 | UNT 40 | Austria | BOKU | Public accession | S |
| 39 | UNT 42 | Austria | UNIBO | BOKU breeding program | S |
| 40 | UNT 43 | Austria | UNIBO | BOKU breeding program | S |
| 41 | UNT 44 | Austria | UNIBO | BOKU breeding program | S |
| 42 | UNT 45 | Germany | UNIBO | Public accession | S |
| 43 | UNT 46 | Russia | UNIBO | Public accession | S |
| 44 | UNT 47 | Austria | UNIBO | Public accession | S |
| 45 | UNT 50 | Germany | USDA | Public accession | S |
| 46 | UNT 52 | Germany | USDA | Public accession | S |
| 47 | UNT 53 | Germany | USDA | Public accession | S |
| 48 | UNT 54 | Germany | USDA | Public accession | S |
| 49 | UNT 55 | Germany | USDA | Public accession | S |
| 50 | UNT 56 | Germany | USDA | Public accession | S |
| 51 | UNT 57 | Germany | USDA | Public accession | S |
| 52 | UNT 58 | Denmark | USDA | Public accession | S |
| 53 | UNT 59 | Austria | USDA | Public accession | S |
| 54 | NA | Spain | CCE | Commercial material | S |
| 55 | NA | Spain | CCE | Commercial material | S |
| 56 | NA | Poland | UPP | Commercial material | W |
| 57 | NA | Germany | KWS | Commercial material | S |
